# Molecular Characterization of a Strawberry *FaASR* Gene in Relation to Fruit Ripening

**DOI:** 10.1371/journal.pone.0024649

**Published:** 2011-09-06

**Authors:** Jian-ye Chen, Du-juan Liu, Yue-ming Jiang, Ming-lei Zhao, Wei Shan, Jian-fei Kuang, Wang-jin Lu

**Affiliations:** 1 State Key Laboratory for Conservation and Utilization of Subtropical Agro-Bioresources/Guangdong Key Laboratory for Postharvest Science, College of Horticultural Science, South China Agricultural University, Guangzhou, The People's Republic of China; 2 South China Botanical Garden, Chinese Academy of Science, Guangzhou, The People's Republic of China; Korea University, Republic of Korea

## Abstract

**Background:**

ABA-, stress- and ripening-induced (ASR) proteins have been reported to act as a downstream component involved in ABA signal transduction. Although much attention has been paid to the roles of ASR in plant development and stress responses, the mechanisms by which ABA regulate fruit ripening at the molecular level are not fully understood. In the present work, a strawberry *ASR* gene was isolated and characterized (*FaASR*), and a polyclonal antibody against FaASR protein was prepared. Furthermore, the effects of ABA, applied to two different developmental stages of strawberry, on fruit ripening and the expression of *FaASR* at transcriptional and translational levels were investigated.

**Methodology/Principal Findings:**

*FaASR*, localized in the cytoplasm and nucleus, contained 193 amino acids and shared common features with other plant ASRs. It also functioned as a transcriptional activator in yeast with trans-activation activity in the N-terminus. During strawberry fruit development, endogenous ABA content, levels of *FaASR* mRNA and protein increased significantly at the initiation of ripening at a white (W) fruit developmental stage. More importantly, application of exogenous ABA to large green (LG) fruit and W fruit markedly increased endogenous ABA content, accelerated fruit ripening, and greatly enhanced the expression of *FaASR* transcripts and the accumulation of FaASR protein simultaneously.

**Conclusions:**

These results indicate that *FaASR* may be involved in strawberry fruit ripening. The observed increase in endogenous ABA content, and enhanced *FaASR* expression at transcriptional and translational levels in response to ABA treatment might partially contribute to the acceleration of strawberry fruit ripening.

## Introduction

Fruits can be classified as climacteric and non-climacteric based on their patterns of respiration and ethylene production during maturation and ripening [1]. Climacteric fruits such as tomato and banana show a burst of ethylene production in association with increased rates of respiration during ripening. In these fruits, the burst of ethylene production is essential as it coordinates the transcription and translation of many ripening-associated genes and downstream proteins responsible for normal fruit ripening [1]–[3]. In contrast, non-climacteric fruits such as pepper, strawberry, and grape exhibit neither the respiratory burst nor ethylene responses like climacteric fruit during ripening [4]–[6]. While there has been intensive research into characterizing the mechanisms of climacteric fruit ripening [1], [7]–[9], there has been relatively less attention paid to the mechanisms involved in the ripening of non-climacteric fruit. Some reports indicate that the ripening of non-climacteric fruits may be related to abscisic acid (ABA), auxin, brassinosteroids, ethylene and methyl jasmonate (MeJA) [4], [5], [10]–[18]. These results suggest that fruit ripening probably involves the integration of multiple hormone signals [19], [20].

There has been considerable research into the role of ABA in both climacteric and non-climacteric fruit ripening. It is well known that the ABA content increases during fruit ripening in both climacteric and non-climacteric fruit [21]–[25]. Moreover, ABA is thought to control ripening through activation of ethylene biosynthesis in climacteric fruits [24] and, thus, ABA can be considered as a candidate for a ripening control factor [24], [25]. Biochemical and genetic evidence has also indicated that ABA biosynthesis and signaling appear to involve a complex network of both positively and negatively regulating components, including kinases, phosphatases and transcriptional regulators [26]–[28]. It has been well documented that 9-cis-epoxycarotenoiddioxygenase (NCED), a key enzyme in ABA biosynthesis, plays an important role in ripening of both climacteric and non-climacteric fruit [19], [24], [25], [29]. In addition, ABA activated the expression of anthocyanin biosynthetic genes and the anthocyanin synthesis-related *VvmybA1* transcription factor [30], [31], and delayed the expression of condensed tannin biosynthetic genes (*VvANR* and *VvLAR2*) in grapes [32]. Recently, proteomic analysis further demonstrated that ABA accelerated grape berry ripening mainly via the regulation of many proteins involved in the ripening process [33]. Unfortunately, many of the cellular components and genes concerning ABA downstream signal transduction have not been well characterized.

ABA-, stress- and ripening-induced (ASR) proteins are hydrophilic charged low-molecular weight plant-specific proteins. The ASR proteins have been shown to be involved in plant development, senescence and fruit ripening, and in response to ABA and abiotic stresses such as water deficit, salt, cold and limited light [34], [35]–[42]. More importantly, grape and tomato ASR proteins have been proposed to regulate the transcription of sugar- and abiotic stress-regulated genes in fruit and vegetative tissues [34], [43], [44], [45]. For examples, grape ASR (VvMSA) could bind to the promoter sequence of a monosaccharide transporter [34] and tomato ASR1 bound coupling element 1 (CE1) of *ABI4* promoter competing with *ABI4*, which produced an ABA insensitive phenotype [43]. These results suggested that the ASR proteins act as a downstream component of a common transduction pathway for sugar and ABA signals. Although several functions of ASRs have been proposed, the precise role of the ASR protein in fruit ripening remains unclear.

Strawberry (*Fragaria* × *ananassa*) is one of the most economically important fresh and processed fruits, consumed for both its pleasant flavour and its nutrient content [46]. However, strawberry fruit have a short shelf-life, mainly due to a rapid loss in firmness and texture [47]. Essential factors that control the growth and development of strawberry fruit are still poorly characterized at the molecular level [48], [49]. The strawberry achenes (a combination of seed and ovary tissue at the base of each pistil) are the true fruit of this specie and are embedded in the epidermal layer of the receptacle connected by vascular bundles [47], [49]. It is well established that fertilized achenes govern the fate of the receptacle and, at least during the early stages of fruit development, auxin synthesized in the achenes promotes the growth of the receptacle [50]. Moreover, auxin negatively regulates almost all strawberry identified ripening-regulated genes [51]–[53]. More recently, gibberellin (GA) has been reported to play a critical role in the development of the strawberry receptacle [49]. However, little attention has been paid to possible roles of other plant hormones in these processes, such as ABA.

In the present work, a strawberry *FaASR* gene was isolated and characterized, and the polyclonal antibody of FaASR was then prepared. The effects of ABA applied to two different developmental stages of strawberry on subsequent fruit ripening with regard to *FaASR* at transcriptional and translational levels were also investigated. The results showed that the ABA-related enhancement in *FaASR* at transcriptional and translational levels may partially contribute to the accelerated ripening of strawberry fruit.

## Results

### Isolation and characterization of full-length cDNA of *FaASR*

One fragment of an *ASR* homologue of approximately 790 bp, was cloned from strawberry fruit by RT-PCR using degenerate primers. The corresponding full-length sequence, designated *FaASR*, was subsequently amplified by RACE-PCR and deposited in GenBank (accession number JN006160). *FaASR* cDNA (989 bp) consisted of a 5′-untranslated region of 65 bp, an ORF of 576 bp and a 3′-untranslated region of 348 bp from the stop codon including poly(A). It encoded the predicted polypeptide (*p*I 6.03) of 192 amino acids (AA), with the predicted molecular weight of 21.05 kDa. FaASR was a small protein rich in Glu (13%), Gly (13%), His (11%) and Ala (9%), which might be a feature of all the ASR proteins [34], [54]. A BLAST search of GenBank revealed that FaASR shared 68% and 65% homology at the protein level with LcASR1 (AAY97997) from tomato and ZmASR1 (ACG35620) from maize, respectively.

**Figure 1:**
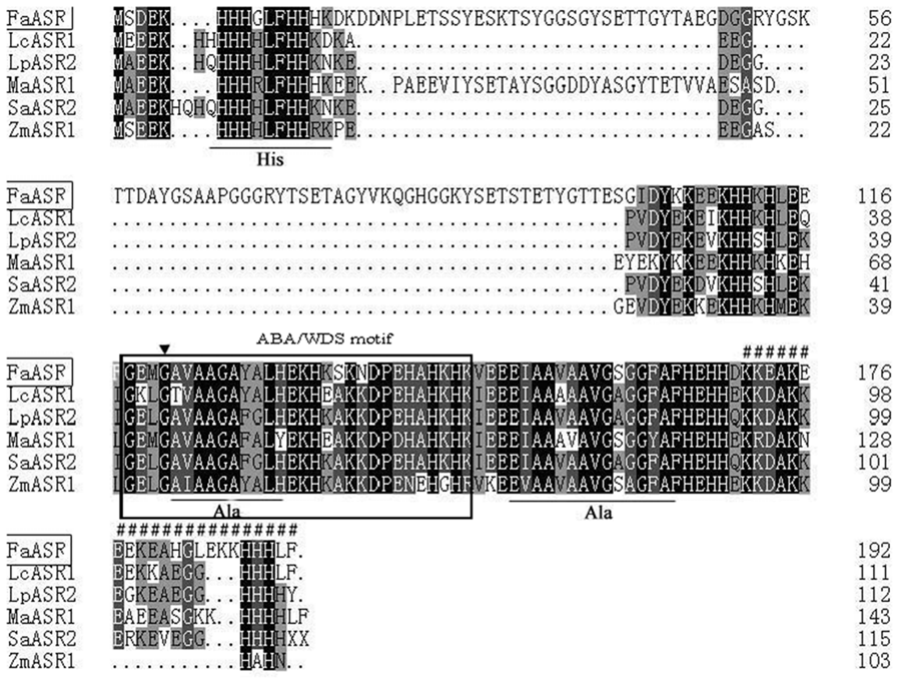
Amino acid sequence alignment of FaASR with other plant ASR proteins. Strawberry FaASR was aligned with tomato LcASR1, LpASR2 and SaASR2, banana MpASR1 and maize ZmASR1. Conserved residues were shaded in black. Gray shading indicated similar residues in 3 out of 6 of the sequences. Gaps were introduced to optimize alignment. Multiple alignments was done by CLUSTALW and viewed with BOXSHADE program, and then manually edited. One Zn-binding His-rich region and two Ala-rich regions were underlined. The ABA/WDS motif was indicated with rectangle. One site for N-myristoylation and a putative nuclear targeting signal (KKEDKEEAEEASGKKHHH) were marked with ‘▾’ and ‘#’, respectively.

The optimal multiple sequence alignment of FaASR protein with its homologies is presented in Figure 1. Although the amino acid sequences of ASR proteins were diverse in both size and composition, two main highly conserved regions existed in the FaASR amino acid sequence: a small N-terminal consensus of 18 to 20 amino acids containing a typical stretch of Zn-binding six His residues in a 10-amino acid sequence, and a longer C-terminal region of at least 80 amino acids exhibiting two Ala-rich regions (Figure 1). A putative nuclear targeting signal (KKEDKEEAEEASGKKHHH) at the C-terminus [34], [54] and one site for N-myristoylation [54] were also found in the FaASR protein. In addition, checking for the specific sequences in FaASR using the BLOCKS method (http://blocks.fhcrc.org) revealed the presence of one ABA/WDS domain (from 118 to 147 AA), which is described in ABA stress- and ripening-induced proteins [55] and in water deficit stress-induced proteins [56]. Overall, these observations indicated that the FaASR shared common features with ASRs obtained from other plants.

### Subcellular localization of FaASR

**Figure 2:**
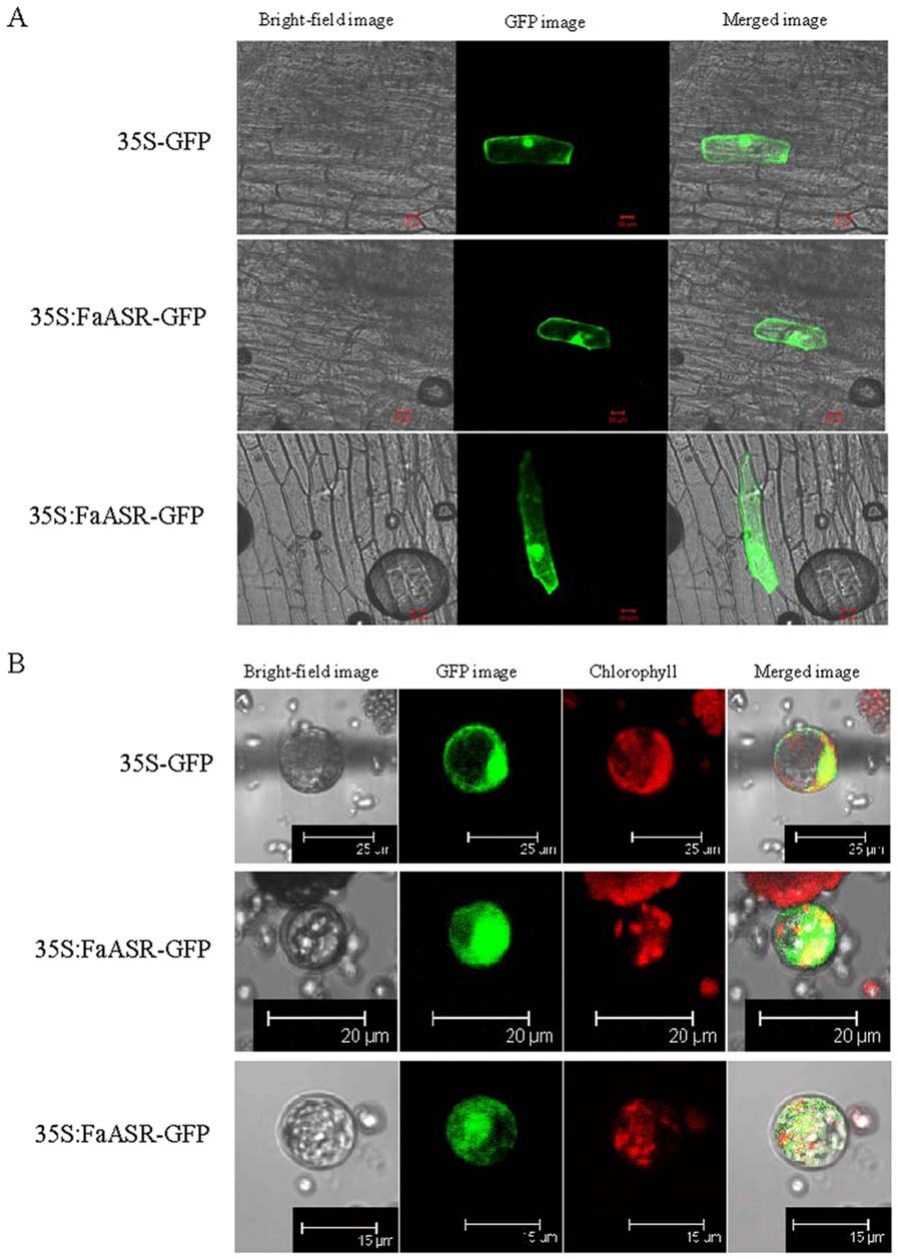
Subcellular localization of FaASR in onion epidermal cells (A) or tobacco protoplasts (B). Constructs carrying GFP or FaASR-GFP were bombarded into onion epidermal cells or transfected into tobacco protoplasts as described in the Materials and methods. GFP and FaASR-GFP fusion proteins were transiently expressed under control of the CaMV 35S promoter and observed with a laser scanning confocal microscope. Experiments were done in triplicate resulting in the same fluorescence pattern, and two different images for FaASR-GFP were presented. The FaASR-GFP fusion protein was present in the cell outlines and the nuclear. The length of Bar was indicated in the photos.

Some reports have shown that ASRs were preferentially localized in the cell nucleus [34], [45], while other reports determined that tomato ASR1 and lily ASR were localized in both cytosol and nucleus compartments by western blot and indirect immunofluorescence analysis [57], [58]. To determine the subcellular localization of FaASR protein, the coding region of *FaASR* was fused in-frame with the *GFP* gene, and the resulting constructs were then bombarded into onion epidermal cells or transfected into tobacco protoplasts. Although the amino acid sequence of FaASR analysis revealed that it contained one nuclear localization sequence (NLS) in its C-terminal region (Figure 1), the fluorescence of FaASR-GFP fusion protein was not exclusively targeted to nuclei in onion epidermal cells (Figure 2A) or tobacco protoplasts (Figure 2B). FaASR-GFP fusion protein was targeted to the cell outlines, likely the cytoplasm, and the nucleus.

### FaASR showed transcriptional activation activity in the N-terminal domain

**Figure 3:**
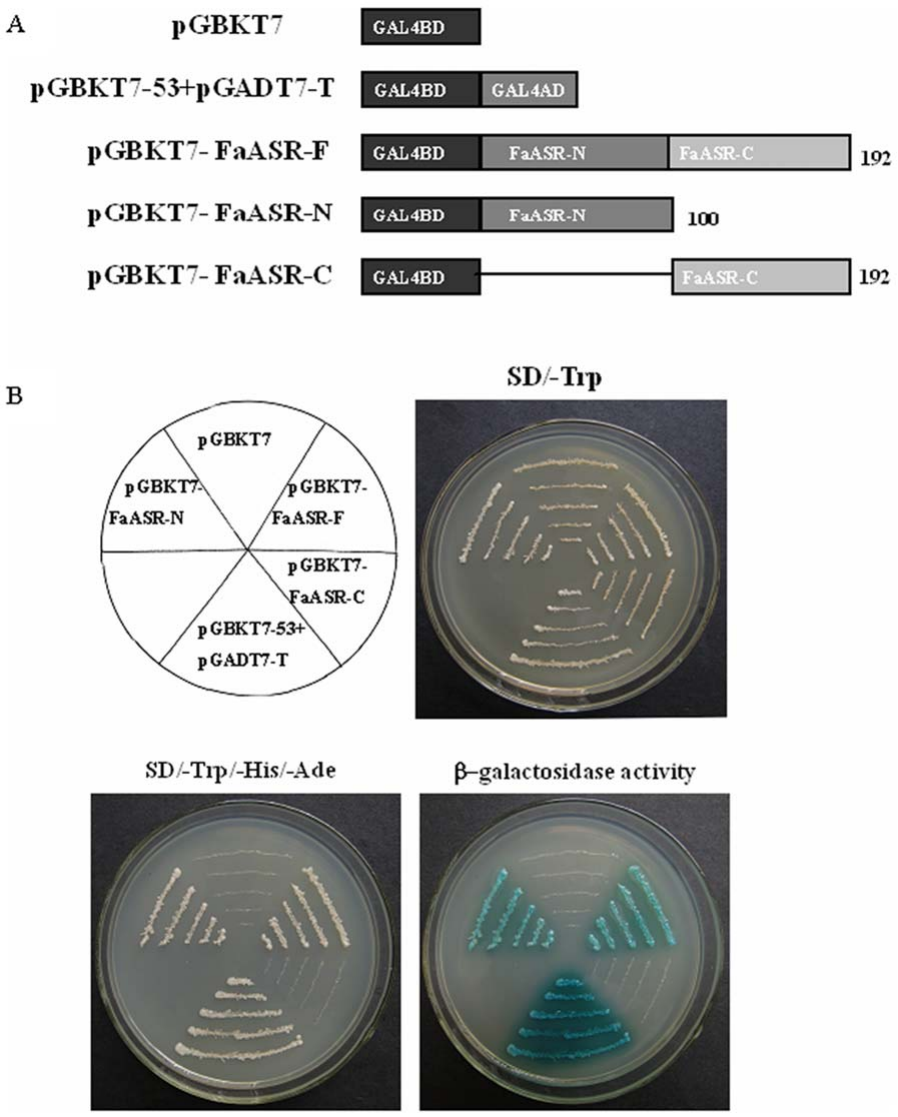
Transcriptional activation of FaASR in yeast. (A) Diagram showing all the reporter constructs for analysis of pGBKT7 (DNA-binding protein)-dependent transactivation of FaASR in the yeast strain AH109. The vectors were constructed with pGBKT7 (negative control), pGBKT7-53 + pGADT7-T (positive control), pGBKT7-FaASR-F (amino acids 1-192), pGBKT7-FaASR-N (amino acids 1–100), and pGBKT7-FaASR-C (amino acids 101–192). (B) Clones transformed with the different vectors were grown on SD plates with or without histidine and adenine for 3 d at 30°C. Transcription activation was monitored by the detection of yeast growth without histidine and adenine and β-galactosidase assay.

To investigate the transcriptional activity of FaASR, a transient expression assay using a GAL4-responsive reporter system was performed. Yeast strain AH109 harboring two reporter genes, *lacZ* and *His3*, was transformed with the fusion plasmids pGBKT7-FaASR-F, pGBKT7-FaASR-N, pGBKT7-FaASR-C, the positive control pGBKT7-53+pGADT7-T, and the negative control pGBKT7, respectively (Figure 3A). Figure 3B showed that the transformed yeast cells harboring pGBKT7-FaASR-F, pGBKT7-FaASR-N and pGBKT7-53+pGADT7-T (the positive control), grew well in the SD medium lacking tryptophan, histidine and adenine, and showed β-galactosidase activity, whereas the cells containing pGBKT7-FaASR-C or pGBKT7 (the negative control) did not show β-galactosidase activity. The data further confirmed that FaASR functioned as a transcriptional activator in yeast, and had trans-activation activity in the N-terminus.

### Induction and purification of the FaASR fusion protein

The ORF sequence of *FaASR* was PCR amplified and digested with *Nde*I and *Bam*HI, which did not cut within the coding region. The resulting PCR fragment was ligated to the *Nde*I/*Bam*HI pre-digested pET-3C vector to generate the recombinant plasmid, pET-3C-*FaASR*. The clones were screened by PCR and subjected to restriction digestion. The inserted fragment was then verified by sequencing to confirm that the *FaASR* coding region was in the correct reading frame with the His-tag in the vector. The confirmed construct pET-3C-*FaASR* was finally transformed into *E. coli* strain BL21 (DE3).

**Figure 4:**
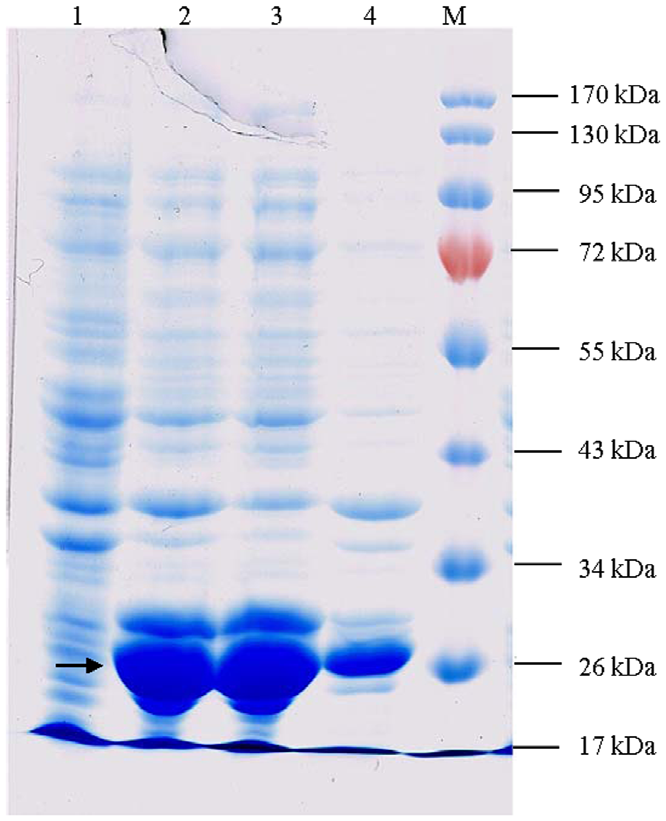
SDS-PAGE analysis of induced and purified recombinant FaASR fusion protein. The gel was visualized by Coomassie Brilliant Blue R250 staining. Lane 1: sample collected before induction by IPTG; Lanes 2–3: samples collected after induction by IPTG for 4 and 6 h, respectively and Lane 4: purified fusion protein. M: protein marker. The arrow showed the protein of recombinant FaASR.

As shown in Figure 4, upon induction by IPTG for 4 or 6 h at 37°C, SDS-PAGE indicated that FaASR was expressed as a major protein product of about 26 kDa in the total cellular protein, when visualized by Coomassie Brilliant Blue R250 staining (Figure 4, Lanes 2 and 3). The molecular weight of the expressed recombinant protein was estimated to be about 26 kDa fused with His-Tag, and therefore the size of expressed pET-FaASR protein was in good agreement with that predicted from the amino acid sequence of FaASR by the bioinformatics method. In addition, the corresponding band was not found in the total cellular protein without IPTG induction (Figure 4, Lane 1). The amount of the recombinant protein was roughly over 60% of the total protein. It was also found that the recombinant protein was expressed in the insoluble fraction of the total bacterial cultures as an inclusion body (data not shown). Similarly, it was also reported that *Ginkgo biloba* GbASR is insoluble when expressed in *E. coli* [54].

Owning to the His-Tag sequence, the recombinant FaASR fusion protein could bind to the Ni-NTA His Bind Resin, and was recovered by eluting with imidazole. The target protein recovered by elution with imidazole had a high degree of purity (>95%) (Figure 4, Lane 4).

### FaASR polyclonal antibody specificity

**Figure 5:**
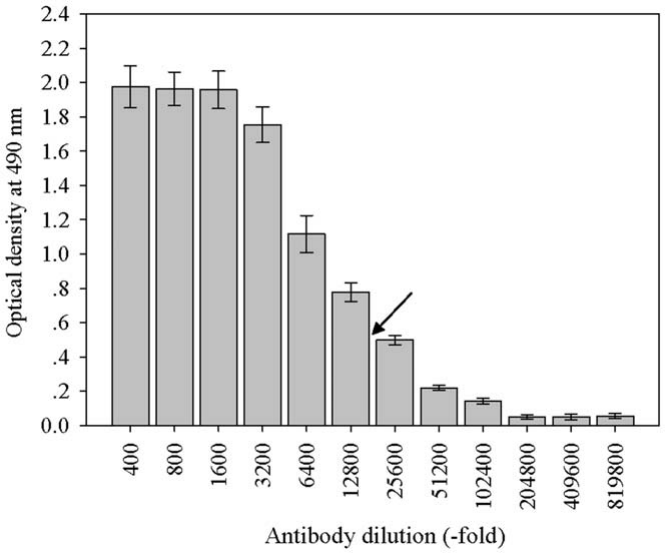
Analysis of anti-FaASR titer by ELISA. The antibody at different dilutions (400- to 819,800-fold) was reacted with the equal amount of the purified fusion protein (1 μg). Antibody titer is defined as the dilution of the antibody corresponding to an absorbance of 0.500 at 490 nm. The arrow indicated the 0.500 absorbance value that corresponds to antibody dilution times of about 25,000. Values in the figure were the means ± S.D. (n = 3).

The immunoglobulin fractions (IgG) against FaASR were purified from rabbit raw antiserum by (NH_4_)_2_SO_4_ precipitation and DEAE-Sepharose A-50 chromatography. The titer of the purified IgG of anti-FaASR was determined by ELISA analysis. The results indicated that the anti-FaASR polyclonal antibody had a high sensitivity, with a 1/25,000 dilution of the anti-FaASR polyclonal antibody being capable of detecting 1 μg of the antigen (Figure 5). In contrast, the pre-immune rabbit serum used as control did not recognize the antigen. Moreover, the affinity and specificity of the obtained FaASR polyclonal antibody was further confirmed against the crude protein extract from strawberry fruit in the following western blot analysis.

### Changes in endogenous ABA content and levels of *FaASR* mRNA and protein at different ripening stages

**Figure 6:**
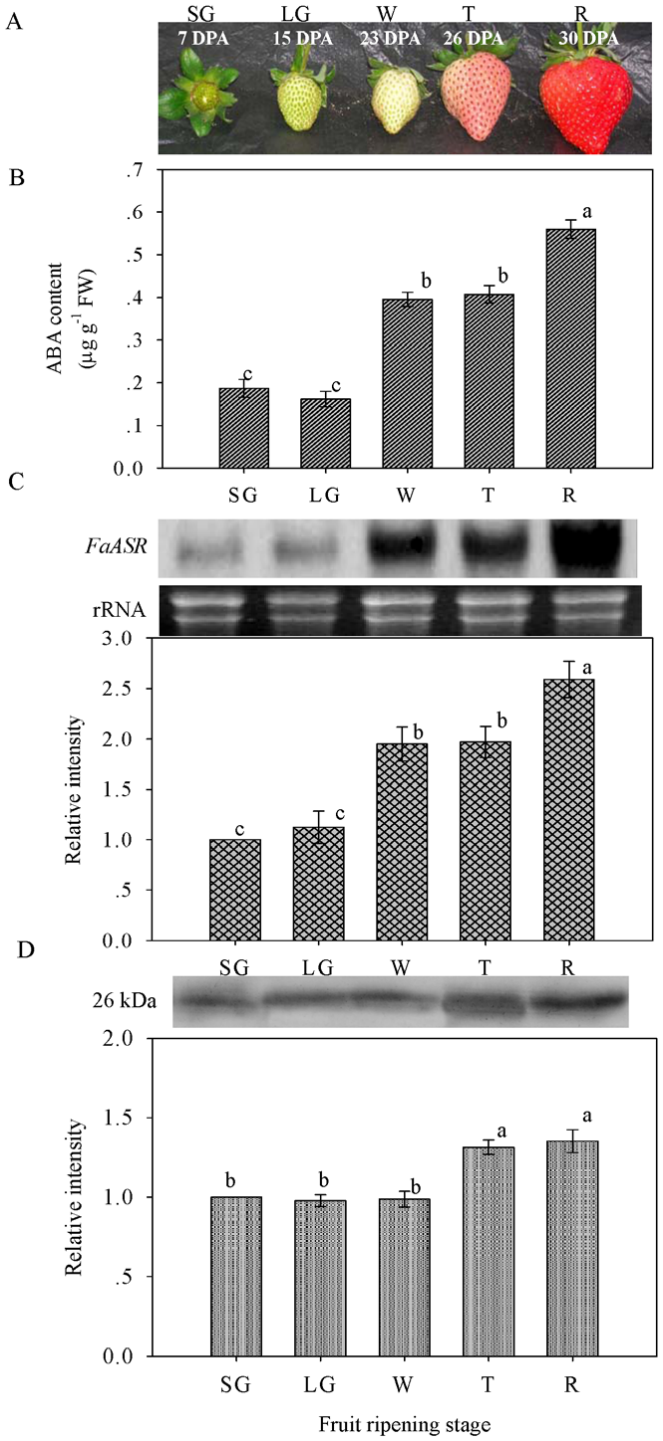
Photograph of fruits representative of each stage (A), SG: small green; LG: large green; W: white; T: turning, 50% red and R: full-ripe red, and changes of endogenous ABA content (B), levels of *FaASR* mRNA (C) and protein (D) at different fruit ripening stages. In (A), each stage of fruit development corresponded to days after post-anthesis (DPA) were indicated. In (C), total RNA (10 μg per lane) was used for northern blot analysis and hybridized with DIG-labeled probe, and ethidium bromide-stained rRNA was shown as the loading control. In (D), equal amounts of protein (30 μg) per lane were subjected to SDS-PAGE and then transferred to a nitrocellulose membrane. Thereafter, the FaASR protein amount was immunodetected by western blot using the anti-FaASR specific polyclonal antibody. In (C) and (D), the quantification of the northern or western blot bands was expressed in relation to the amount in fruit sampled at SG, which was set to 1. In (B), (C) and (D), vertical bars represented standard deviations (SD) of means (n = 3). Different letters indicated a statistical difference at the 5% level among data groups according to the Duncan's multiple range test.

To investigate whether the changes in endogenous ABA content and levels of *FaASR* mRNA and protein in strawberry were associated with fruit ripening, the endogenous ABA content and temporal expression patterns of *FaASR* mRNA and protein were analyzed at the five different developmental stages, from small green to ripening fruit. As shown in Figure 6, endogenous ABA content (Figure 6B), levels of *FaASR* mRNA (Figure 6C) and amounts of FaASR protein (Figure 6D) were lowest in the small green (SG) and large green (LG) fruit with higher fruit firmness [51], [59]. When ripening commenced at white (W), turning (T) and full-ripe red (R) developmental stages, fruit firmness decreased remarkably [59] once the red fruit skin colour developed (Figure S1A), in association with an increase in endogenous ABA content, transcripts of *FaASR*, and amounts of FaASR protein at W or T stage, and remained at high levels at the late ripening stages (Figure 6). In addition, *FaNCED* mRNA accumulated at W or T stage, and reached highest levels at the late ripening stages (Figure S1B). This trend was consistent with the accumulation of endogenous ABA content, *FaASR* mRNA and FaASR protein. These results indicated that endogenous ABA content and levels of *FaASR* mRNA and protein might be associated with the initiation of strawberry fruit ripening.

### Effect of exogenous ABA treatment on endogenous ABA content and fruit ripening

**Figure 7:**
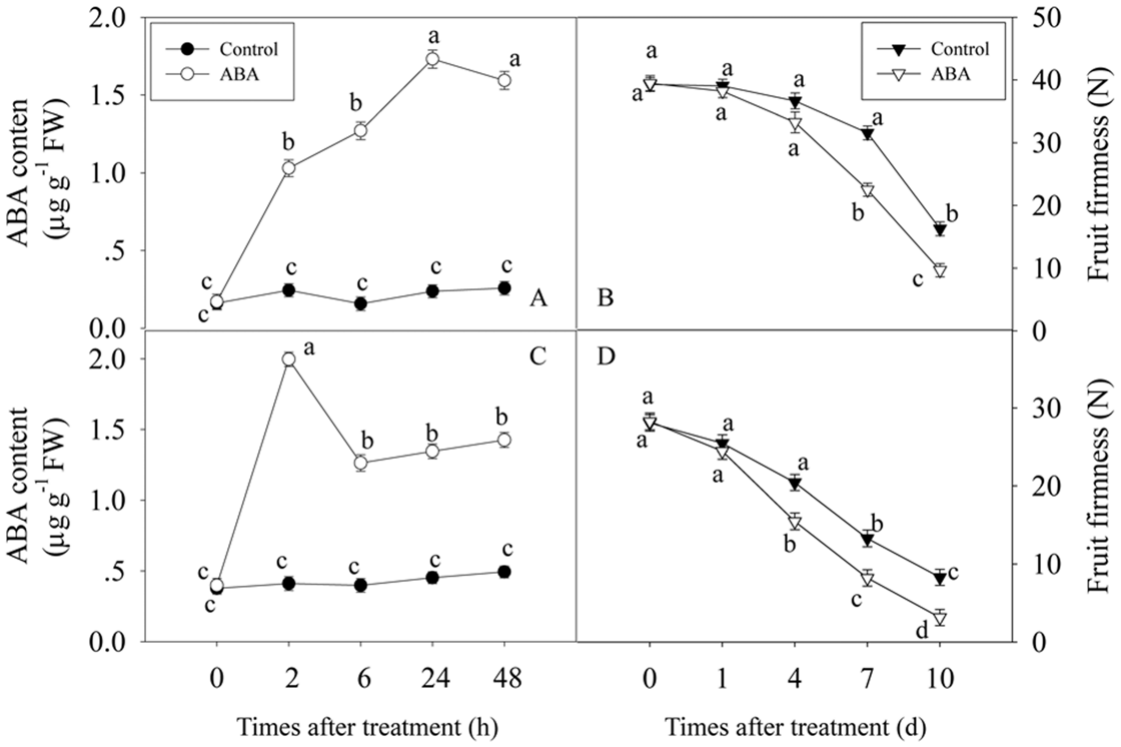
Changes of endogenous ABA content (A and C) and fruit firmness (B and D) after ABA treatment. (A) and (B) showed changes of endogenous ABA content and fruit firmness at the LG stage (about 15 days after post-anthesis (DPA)), while (C) and (D) presented changes of endogenous ABA content and fruit firmness at the W stage (about 23 DPA), respectively. Selected fruit were dipped for 1 min in a solution containing 0 (control) or 100 µM ABA, and then sampled at 0, 0.5, 2, 6, 12 h, 1, 2, 4, 7 and 10 days. Each value represented the means of three replicates, and vertical bars indicated the SD. Different letters indicated a statistical difference at the 5% level among data groups according to the Duncan's multiple range test.

To further confirm the role of ABA involved in fruit ripening, exogenous ABA was applied to the fruit at two different developmental stages and then endogenous ABA content and fruit firmness were analyzed. When ABA was applied to the fruit at LG stage, endogenous ABA content in the ABA-treated fruit increased rapidly after 2 h of treatment, and steadily increased to about 6.47- and 6.21-fold higher than that in the control fruit at 6 and 24 h, respectively (Figure 7A). In addition, fruit firmness of the ABA-treated fruit was lower than that of control fruit, with the fruit firmness of the ABA-treated fruit at 7 and 10 d after treatment being 39.0% and 30.0% lower than that of control fruit, respectively (Figure 7B), indicating that ABA also accelerated fruit ripening and softening. ABA treatments also resulted in greater red colour development (Figure S2). Similar patterns in endogenous ABA content, fruit firmness and red colour development were also observed in the fruit when exogenous ABA was applied at W stage (Figure 7C and 7D, Figure S2). Moreover, ABA treatment at LG or W stage greatly enhanced the accumulations of *FaNCED* mRNA (Figure S3), which confirmed that the increase in ABA content was mainly due to endogenous ABA biosynthesis. These results indicated that strawberry fruit ripening was closely related to ABA accumulation, that is, ABA may play an important role in regulating fruit ripening.

### Effect of exogenous ABA treatment on the accumulations of *FaASR* mRNA and protein

**Figure 8:**
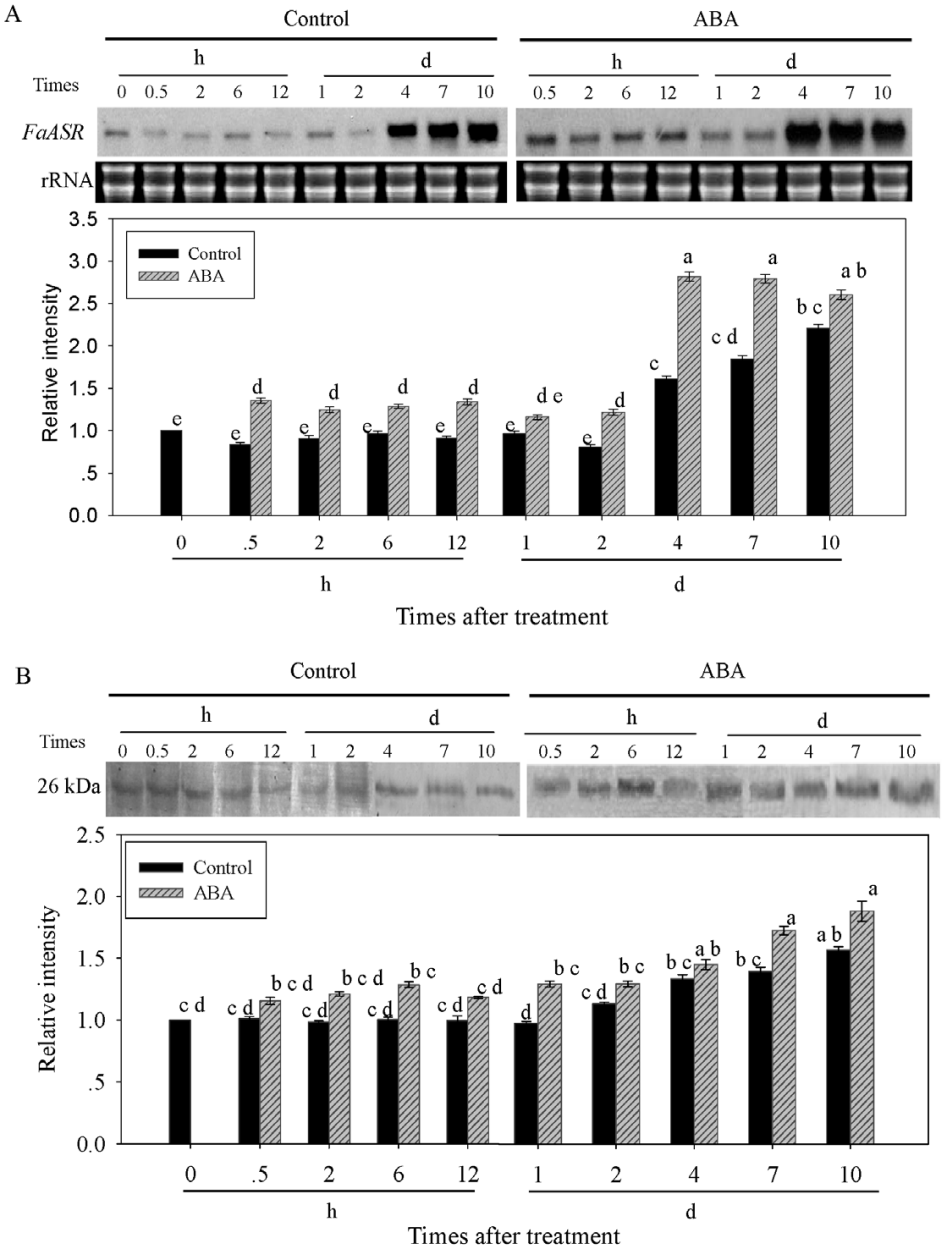
Changes in *FaASR* mRNA (A) and protein (B) accumulations after ABA treatment at the LG stage. Selected fruits at the LG stage (about 15 days after post-anthesis) were dipped for 1 min in a solution containing 0 (control) or 100 µM ABA, and then sampled at 0, 0.5, 2, 6 and 12 h, 1, 2, 4, 7 and 10 days. In (A), total RNA (10 μg per lane) was used for northern blot analysis and hybridized with DIG-labeled probe, and ethidium bromide-stained rRNA was shown as the loading control. In (B), equal amounts of protein (30 μg per lane) were subjected to SDS-PAGE and then transferred to a nitrocellulose membrane. Thereafter, the FaASR protein amount was immunodetected by western blot using the anti-FaASR specific polyclonal antibody. The quantification of the northern or western blot bands was expressed in relation to the amount in control fruit sampled at time 0, which was set to 1. Vertical bars represented standard deviations (SD) of means (n = 3). Different letters indicated a statistical difference at the 5% level among data groups according to the Duncan's multiple range test.

The expression of *FaASR* at mRNA and protein levels after application of exogenous ABA at LG and W stages were analyzed, to further confirm the possible association of *FaASR* mRNA and its protein accumulation with exogenous ABA-accelerated fruit ripening. As shown in Figure 8, when ABA was applied to fruit at the LG stage, *FaASR* mRNA transcripts (Figure 8A) and protein (Figure 8B) began to increase within 0.5 h and 2 h after ABA treatment, and rose to a peak at about 4 d and 10 d, with 1.75- and 1.21-fold higher abundance than those in the control fruit, respectively.

**Figure 9:**
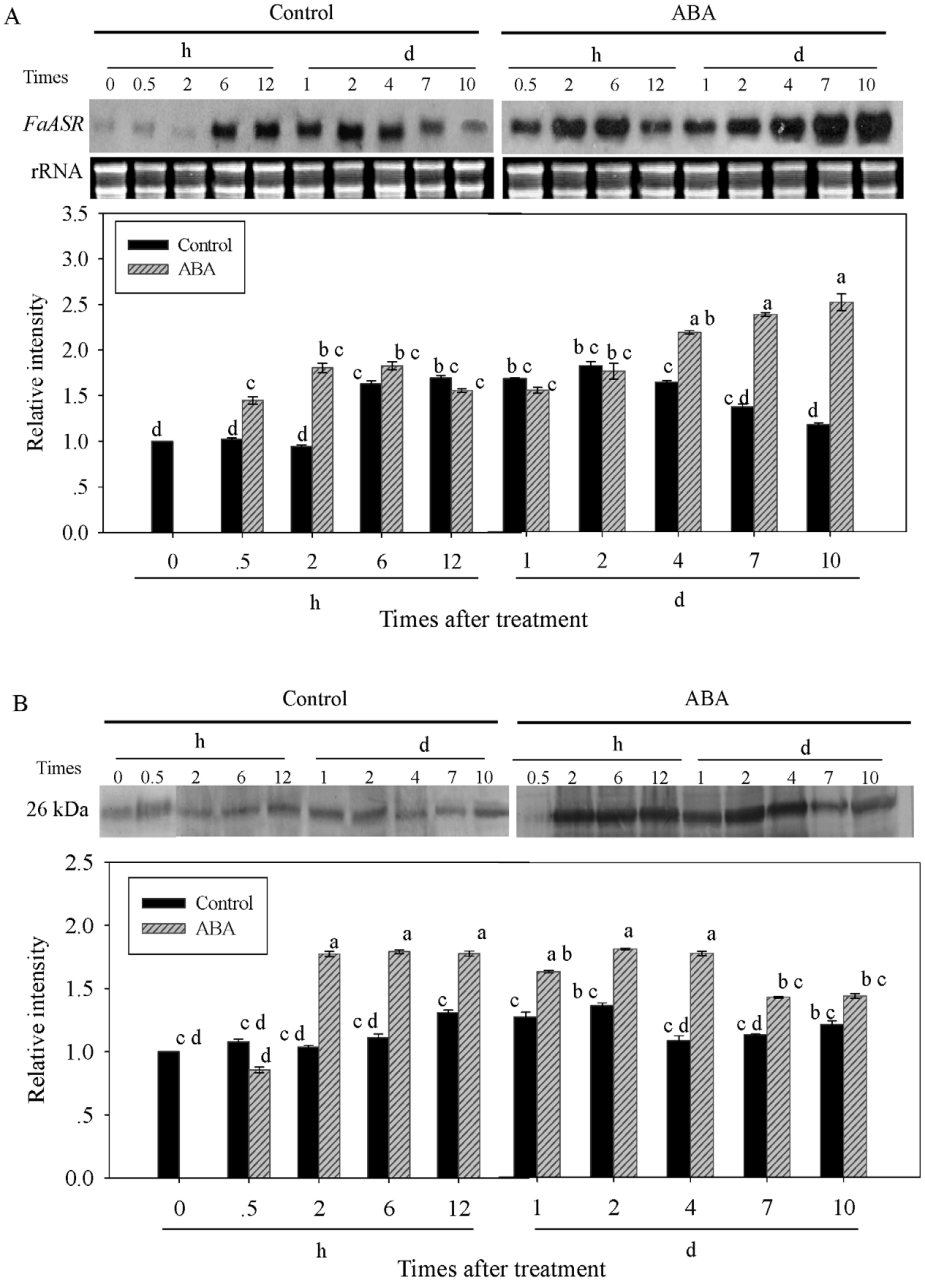
Changes in *FaASR* mRNA (A) and protein (B) accumulations after ABA treatment at the W stage. Selected fruits at the W stage (about 23 days after post-anthesis) were dipped for 1 min in a solution containing 0 (control) or 100 µM ABA, and then sampled at 0, 0.5, 2, 6 and 12 h, 1, 2, 4, 7 and 10 days. In (A), total RNA (10 μg per lane) was used for northern blot analysis and hybridized with DIG-labeled probe, and ethidium bromide-stained rRNA was shown as the loading control. In (B), equal amounts of protein (30 μg per lane) were subjected to SDS-PAGE and then transferred to a nitrocellulose membrane. Thereafter, the FaASR protein amount was immunodetected by western blot using the anti-FaASR specific polyclonal antibody. The quantification of the northern or western blot bands was expressed in relation to the amount in control fruit sampled at time 0, which was set to 1. Vertical bars represented standard deviations (SD) of means (n = 3). Different letters indicated a statistical difference at the 5% level among data groups according to the Duncan's multiple range test.

When ABA treatment was applied to fruit at the W stage, the abundance of *FaASR* mRNA increased within 0.5 h. *FaASR* transcripts reached a peak at 10 d after the treatment that was 1.15-fold higher than that in the control fruit (Figure 9A). Meanwhile, the amount of FaASR protein in the ABA-treated fruit also increased significantly within 2 h (1.71-fold higher than that in the control fruit), and remained at a higher level until 4 d after the treatment before decreasing (Figure 9B). These results indicated that ABA treatment could induce the expression of *FaASR* at both transcriptional and translational levels.

## Discussion

To date, more than 20 *ASR* orthologs have been cloned from various species of dicotyledonous and monocotyledonous plants [60], including *Cucumis melo* [38], lily [61], grape [34], *Ginkgo biloba* [54], potato [62], maize [63], rice [64], pummelo [55] and loblolly pine [56]. However, no *ASR*-like genes have been identified in *Arabidopsis* [34], [41]. In the present work, a novel *ASR* gene, *FaASR*, was isolated and characterized from strawberry fruit for the first time. *FaASR* displayed sequence similarity to all previously characterized ASR sequences (Figure 1) and shared the main characteristics of ASR proteins. FaASR was deduced to encode a polypeptide of 192 amino acids with a predicted molecular weight of 21.05 kDa, which was in good agreement with that of the recombinant FaASR expressed in *E. coli*. Furthermore, the protein sequence contained two highly conserved ASR protein regions, a nuclear targeting signal and an ABA/WDS motif (Figure 1). Transcriptional activation analysis showed that FaASR could activate transcription in yeast cells and the activation activity was mapped to its variable N-terminal region (Figure 3B). These data allow us to conclude that *FaASR* belongs to the *ASR* family and may function as a transcription activator. Similarly, other reported ASR proteins are suggested to function as transcription factors that likely target hexose transporters and ABA responsive genes [34], [43], [45].

Subcellular localization of FaASR-GFP in onion epidermal cells or tobacco protoplasts indicated that the FaASR-GFP fusion protein was not exclusively targeted to nuclei (Figure 2), although one nuclear localization sequence (NLS) existed in its C-terminal region (Figure 1). Many nuclear proteins are reportedly also found in the cytoplasm, including tomato ASR1 and lily ASR [57], [58], [65] and translocation of proteins between the two compartments might be used to regulate the activity of such proteins. Alternatively, one protein might display a distinct role in each compartment [66]. In addition, molecules with a mass of up to 40–60 kDa are capable of diffusing passively through nuclear pores along their concentration gradient, whereas others are actively transported by utilizing nuclear localization signals that are recognized by receptor molecules which dock with their cargo at the nuclear pore [65]. Recently, Urtasun *et al.* reported that tomato ASR1 showed predominantly cytoplasmic localization in yeast using indirect immunofluorescence with ASR1-specific antiserum [67]. The observed cytoplasmic localization is a long recognized feature of steroid hormone-receptor transcription factors, but is quite intriguing for non-receptor proteins like ASR. In addition, the observed cytoplasmic nature of ASR1 in the present study is also consistent with recent evidence showing that the tomato ASR1 could function as a chaperone [68].

*ASR* genes have been suggested to play important roles in plant responses to developmental and environmental signals, including senescence, fruit ripening, pollen maturation and glucose metabolism [34], [38], [44], [45], [61], [62], [69]. In addition, *ASR* genes are typically upregulated by ABA and in response to abiotic and biotic stresses [34]-[36], [39], [41], [42], [57]. Moreover, the ASR protein acts as a downstream component of a common signal transduction pathway for sugar and ABA signals [34], [37], [43]. It is clear that ABA is implicated in many processes including fruit ripening [1], [2], for example, ABA can hasten the initiation of ripening of tomato fruit and grape berry [19], [20], [24], [25], [33]. In non-climacteric fruits such as sweet berry, grape berry and orange, the ABA content gradually accumulates with sugar accumulation and fruit ripening [19], [21], [23], [25]. ABA has also been reported to accelerate sucrose uptake, advance colour development and increase PAL activity in strawberry fruit and cortex discs [70]–[72].

The molecular mechanism of ABA action and its role in regulating *FaASR* expression during strawberry fruit ripening are still not well understood. In the present work, endogenous ABA content, levels of *FaASR* mRNA and protein significantly increased at the beginning or after ripening initiation (W developmental stage) (Figure 6). Application of exogenous ABA at LG and W fruit developmental stages increased the endogenous ABA content and accelerated fruit ripening (Figure 7). While the peak of endogenous ABA content was recorded at 2 h after commencing ABA treatment, it might just represent exogenous ABA absorbed by the fruit (Figure 7). More importantly, enhanced accumulations of *FaNCED* mRNA at 6–48 h after ABA treatment confirmed that the increase in ABA content was mainly due to endogenous ABA biosynthesis (Figure S3). Furthermore, ABA treatment at these two developmental stages enhanced the expression of *FaASR* at transcriptional and translational levels (Figures 8 and 9) simultaneously, indicating that FaASR was also upregulated by ABA. These results further confirmed the hypothesis that ASR acts as a component of the transduction pathway for ABA signaling and is involved in non-climacteric fruit ripening, as reported in grape berry [34], and also in climacteric fruit such as tomato [44], [60]. Our data represent the response of whole strawberry fruit (receptacle plus achenes) [18], [46], [73], which differs from numerous other studies whereby the fleshy receptacle and achenes have been evaluated separately [12], [49], [74], [75]. It has been shown that free auxin levels peak in the receptacle and achenes prior to the white stage and subsequently decline as the fruit matures. The coordinated action between achene and receptacle is part of the mechanism ensuring achene maturation prior to fruit ripening [4], [50]. More recently, Csukasi *et al*. [49] revealed key elements of GA responses in strawberry and pointed to a critical role for GA in the development of the receptacle. In addition, Archbold and Dennis [76] reported that ABA was present in both strawberry receptacle and achenes, with total quantities in both achenes and receptacle increasing during fruit ripening, and Perkins-Veazie [47] suggested that the change in the auxin to ABA ratio in achenes and receptacle may be all that was needed to shift the genetic message from growth to ripening. Therefore, during strawberry fruit ripening, the possible coordinated action between achenes and the receptacle in relation to ABA and *FaASR* need to be further elucidated.

This study further highlights that the role of hormones in the control of strawberry ripening appears to be quite complex. Earlier studies on strawberry fruit ripening have shown that other plant growth regulators such as auxins [50], [51], gibberellic acid (GA_3_) [49], [77], ethylene [16], nitric oxide (NO) [78] and methyl jasmonate (MeJA) [18] could also modulate fruit ripening. In addition, it has been proposed that the accelerated changes in colour development and softening of strawberry produced by ABA treatment can be attributed to ethylene [70], but contradictory results were obtained by Bustamante et al. [79]. Thus, the interactions among these hormones in relation to the initiation and progression of fruit ripening need to be further investigated to determine their relative contributions in non-climacteric fruit.

### Conclusions

In the present study, the strawberry fruit *ASR* gene *FaASR* was isolated and characterized, and its polyantibody was also prepared. Moreover, the enhanced expressions of *FaASR* at transcriptional and translational levels by ABA treatment suggest that this protein might partially contribute to the acceleration of strawberry fruit ripening. Further experiment involving a transgenic approach merits consideration as a way to advance our understanding of the role of *FaASR* in regulating fruit ripening.

## Materials and Methods

### Ethics statement

All rabbits were raised under standardized pathogen-free conditions in the College of Veterinary Medicine at South China Agricultural University. The study protocol for the experimental use of the animals was approved by the Ethics Committee of South China Agricultural University.

### Plant materials

Strawberry plants (*Fragaria × ananassa*, cv. ‘Toyonaka’) were grown under field conditions at the South China Agricultural University, Guangzhou, China. Fruits at different developmental stages were harvested, transferred to the laboratory and then classified by the size and skin color into five developmental stages as described by [51]: small green (SG, about 7 days after post-anthesis (DPA)), large green (LG, about 15 DPA), white (W, about 23 DPA), turning (T, 50% red, about 26 DPA), and full-ripe red (R, about 30 DPA). The calyx and peduncle were removed, and the whole fruit (receptacle plus achenes) was collected. The samples were washed in distill water, drained, frozen in liquid nitrogen and stored at −80°C prior to analysis.

### ABA treatment

Strawberry fruit at LG and W stages were randomly selected on the basis of size and absence of physical damage. The selected fruit were tagged in preparation for treatment with 100 µM ABA in 0.5% (v/v) Tween 20 or 0.5% (v/v) Tween 20 (control). Powdered [±] *cis*, *trans*-ABA (Sigma-Aldrich, Inc., St. Louis, MO, USA) was first dissolved in 1 ml of 70% ethanol and then diluted to a final volume of 1 l with 0.5% (v/v) Tween 20. Each fruit was dipped for 1 min. After 0, 0.5, 2, 6, 12 h, 1, 2, 4, 7 and 10 days of each treatment, 50 randomly selected fruit including the achene and receptacle were collected, washed with 70% ethanol, rinsed twice with 0.5% (v/v) Tween 20 to reduce the residual ABA on the fruit surface, and then frozen in liquid nitrogen and stored at −80°C until use.

### Measurement of fruit surface color and firmness

Surface colour for individual fruit was measured using a reflectance colorimeter (Chromameter-2 Re-flectance, Minolta, Japan) equipped with a CR-300 measuring head. For each fruit, the color was measured in three different zones of its surface, and the L*, a* and b* colour space data values were recorded, of which parameter b* is a useful index for strawberry fruit ripeness [70]. Fruit firmness was measured using a digital force gauge pressure tester (Model Instron 5542, INSTRON Co., USA), using a 4 mm cylinder tip. Two measurements on each equatorial side were performed on each fruit. Twenty five fruit per sample were measured and the mean was recorded and expressed as Newtons (N) ± standard deviations (SD).

### Determination of ABA content

Quantitative determination of ABA content was performed following the protocol described by [80] with minor modifications. Briefly, frozen fruit (5 g) was homogenised in cold (4°C) 80% methanol containing 100 µg butylated hydroxytoluene, and 324 ng D_3_-ABA (Roger Horgan, Garden City, NY, USA) as the internal standard. The mixture was stirred overnight at −20°C. After filtration, the extract was adjusted to pH 8.0 with ammonia and reduced to the aqueous phase under vacuum using a rotary film evaporator (RFE) with a water bath at 35°C. The aqueous phase was sealed in a test tube, frozen at −20°C, melted and thawed three times at 20°C. After intermittent centrifugation (1200 *g* for 20 min), polyvinylpolypyrrolidone (1 mg) was added to the supernatant. The combined solution was homogenised at 4.5 m/s for 10 s in a 10 ml sealed test-tube and then set aside for 10 min. Then, the samples were filtered through a layer of filter paper prewetted with 80% methanol. The filtrate was collected and adjusted to pH 2.5–3.0 with 2 M acetic acid. The filtrate was extracted three times in 5 ml of ethyl acetate. The organic layers were combined and evaporated to dryness under vacuum using a RFE with a water bath at 35°C. Extracts were dissolved in 5 ml 0.1 M acetic acid and passed through a C_18_ Waters Sep-Pak cartridge (Waters, Milford, MA, USA) for purification. The methyl esters of the purified fraction were prepared by dissolving the residue in 1 ml methanol and adding 1.5 ml ethereal diazomethane. The excess diazomethane was removed under a stream of dry oxygen-free nitrogen gas. The methylated samples were redissovled in ethyl acetate before being analysed by gas chromatography-mass spectrometry (GC-MS). The injection volume was 1 µl. The experiment was repeated three times. GC-MS was conducted using an Agilent 6890/5973 GC-MS system (Agilent, Santa Clara, CA, USA).

### RNA extraction, isolation of strawberry *ASR* full length cDNA

Total RNA from whole strawberry fruit (receptacle plus achenes) was extracted using the hot borate method of [81]. Frozen tissues (10 g) were ground to a fine powder in a mortar using a pestle in the presence of liquid nitrogen. The extracted total RNA was used as templates for RT-PCR. The product (the first-strand cDNA) was subjected to PCR amplification. Degenerate primers of *ASR* (i.e. sense: 5′-GAGCACCACCACCACCANYTNTTYCAYC-3′ and antisense: 5′-CCTCCTTGGCCTCCTTCTTYTCRTGRTG-3′, where Y is C or T, R is A or G, and N is all four nucleotides) were designed with reference to the conserved amino acids sequences of *ASR*. Reactions for the RT-PCR were subjected to one cycle of 94°C for 3 min, 35 cycles each at 94°C for 1 min, 45°C for 2 min and 72°C for 2 min, and then one cycle of 72°C for 10 min. PCR products of the predicted size (about 790 bp in length) were purified and cloned into pGEM-T easy vector (Promega, Madison, WI, USA). The nucleotide sequences of the cDNA inserts were determined using the thermo sequenase dye terminator cycle sequencing kit and a 3730 DNA sequencer (PerkinElmer Applied Biosystems, Foster City, CA, USA).

Consequently, 3′- or 5′- rapid amplification of cDNA ends (3′- or 5′-RACE-PCR) was performed using cDNA amplification kits (TaKaRa, Shiga, Japan) according to the manufacturer's protocol. In order to amplify 3′-end and 5′-end fragments, the specific primers for *FaASR* (3′-RACE: outer, ATCCGTTAGAAACCTCCTCC, and inner ATCCGTTAGAAACCTCCTCC; 5′-RACE: outer, CCTCCTTGGCCTCCTTCTTGTCATGCTG, middle, GCGGCGATCTCCTCCTCGAGATTCTG, and inner, GCTCCATGTGCTTGTGGTGCTTATCGTCCT) were designed based on the nucleotide sequences of the cDNA fragments already cloned by RT-PCR. The 3′- and 5′- RACE-PCR products were cloned and sequenced as described above.

### Bioinformatics analysis

Identification of nucleotide sequences from RT-PCR clones were established using the NCBI Blast program [http://www.ncbi.nlm.nih.gov/BLAST]. Alignment and comparison of sequence were made using the ClustalW program (http://www.ebi.ac.uk/clustalw). Open reading frame and protein prediction were made using NCBI ORF Finder [http://www.ncbi.nlm.nih.gov/gorf/gorf.html]. The theoretical isoelectric point (p*I*) and mass values for mature peptides were calculated using the PeptideMass program [http://us.expasy.org/tools/peptide-mass.html].

### Subcellular localization analysis

The coding region of the *FaASR* gene was amplified by PCR and inserted into the *EcoR*I and *Sal*I sites of the pEZS-NL-GFP vector to generate FaASR-GFP in-frame fusion protein. Two systems of living plant cells, onion epidermal cells and tobacco protoplasts, were used for subcellular localization. For onion, the fusion construct or the control GFP vector were introduced into epidermal cells by particle bombardment using a Bio-Rad (Hercules, CA, USA) Biolistic PDS 1000/He system. For tobacco, protoplasts used for transfection were isolated from tobacco leaves of 3-to 4-week-old plants according to the method described by [82]. Protoplasts were transfected by a modified polyethylene glycol method as described by [83]. GFP fluorescence was observed with a laser scanning confocal microscope. All transient expression assays were repeated at least three times.

### Transcriptional activation analysis in yeast cells

The transcriptional activation activity of FaASR was determined in yeast cells. The entire or partial coding regions of FaASR were obtained by PCR using primers: 5′-GGAATTCATGTCTGACGAGAAGCACCACCAC-3′ and 5′- GCGTCGACGGAAGAGATGATGGTGCTTC-3′, for the entire FaASR; 5′- GGAATTCATGTCTGACGAGAAGCACCACCAC-3′ and 5′- GCGTCGACGAGACTCGGTGGTGCCATAAG-3′, for the N-terminus; 5′- GGAATTCGGAATCGATTACAAGAAGGAGG-3′ and 5′- GCGTCGACGGAAGAGATGATGGTGCTTC-3′, for the C-terminus. The PCR products were inserted into the *EcoR*I/*Sal*I sites of the pGBKT7 vector to create the pGBKT7-FaASR-F, pGBKT7-FaASR-N, pGBKT7-FaASR-C construct, respectively. According to the protocol of the manufacturer (Clontech, Palo Alto, CA, USA), the three constructs, the positive control pGBKT7-53+pGADT7-T, and the negative control pGBKT7 plasmids were used to transform the AH109 yeast strain. The transformed strains were streaked onto synthetic dextrose (SD)/-Trp or SD/-Trp-His-Ade plates. The trans-activation activity of each protein was evaluated according to their growth status and β-galactosidase activity.

### Preparation of FaASR polyclonal antibody

Protein expression and purification: *FaASR* was cloned into the *Nhe*I and *Bam*HI sites of the pET-3C vector. The sequenced His-FaASR construct was introduced into *E. coli* BL21 (DE3) for expression. Fusion proteins were expressed in BL21 (DE3) cells by adding 1.0 mM isopropyl-β-D-thiogalactoside (IPTG, Sigma-Aldrich) to the culture medium for 4 or 6 h at 37°C. The expression levels of the His-FaASR fusion proteins were assessed by analyzing total protein on SDS-PAGE followed by Coomassie Brilliant Blue R250 staining, and then purified using Ni-NTA agarose (Qiagen, Hilden, Germany) according to manufacturer's manual.

The FaASR antibody was generated in a New Zealand rabbit. Initially, 0.5 g of the purified FaASR protein was injected (subcutaneous) into a rabbit after being emulsified with Freund's complete adjuvant. Four booster injections were given at a 10-day interval, and the antiserum was collected 10 days after the last injection. Rabbit IgG was purified by precipitation with 50% saturated (NH_4_)_2_SO_4_ and DEAE-Sepharose column chromatography. The antibody titer was measured using an enzyme-linked immunosorbent analysis (ELISA) according to the methods described by [84].

### Northern blot analysis

Total RNA (10 µg) was separated on a 1.2% agarose-formadehyde gel and capillary blotted onto positively-charged nylon membrane (Biodyne® B, 0.45 µm, PALL Co. Sarasota, FL, USA). The RNA was fixed to the membrane by baking for 2 h at 80°C and then cross-linked to the membranes using an ultraviolet cross linker (Amersham Biosciences, Piscataway, NJ, USA). The membranes were prehybridized for more than 3 h in SDS buffer containing 50% deionized formamide (v/v), 5 × SSC, 7% SDS, 2% blocking reagent (Roche Diagnostics, Mannheim, Germany), 50 mM sodium-phosphate (pH 7.0) and 0.1% N-lauroylsarcosine (w/v). Hybridization was performed overnight in the same buffer containing the *FaASR* gene-specific digoxin (DIG)-labeled probe at 45°C. The probe was prepared with a DIG probe synthesis kit (Roche Applied Science, Mannheim, Germany) according to the manufacturer's instructions. The probe was synthesized from the 3′-untranslated regions of the *FaASR* gene (i.e. DIG-For: 5′- GCGAGACCTCCACTGAAACT-3′ and DIG-Rev: 5′- GCAAACCCAGATAATACCCA-3′). Following hybridization, membranes were washed twice for 10 min with 2 × SSC containing 0.1% SDS at 25°C, followed by washing twice for 30 min in 0.1 × SSC containing 0.1% SDS at 62°C. The chemiluminescence signals were detected using CDP-Star ™ (Roche Diagnostics) as described by the manufacturer. Membranes were scanned with a densitometer (Bio-Rad Fluor-S Multimager) and quantified using Bio-Rad Quantity One software in at least three independent hybridization signals.

### Protein extraction and western blot analysis

Protein from ground fruit samples was extracted with phenol and purified by ammonium acetate-methanol precipitation as described by [85]. The protein concentration in all extracts was determined using the RC/DC protein assay kit (Bio-Rad Laboratories, Hercules, CA) to compensate for interfering compounds according to the manufacturer's protocol using bovine serum albumin (BSA) as standard. Separation of proteins was performed by SDS-PAGE using 30 µg protein per lane. After electrophoresis, protein was electro-transferred onto poly-vinylidene fluoride (PVDF) membrane (0.22 µm, Bio-Rad) using a Bio-Rad transfer apparatus. After rinsing in TBS buffer [10 mM Tris–HCl (pH 7.5) and 150 mM NaCl], the membrane was blocked with 3% (w/v) BSA in TBS containing 0.05% (v/v) Tween 20 for 2 h and then incubated with gentle shaking for 3 h in 5000-fold diluted primary FaASR polyclonal antibody at 25°C. Following extensive washes with TBST buffer [TBS, 0.05% (v/v) Tween 20], the membrane was incubated with goat anti-rabbit IgG-alkaline phosphatase conjugate (1∶2000 diluted in TBST) (Promega, Madison, WI, USA) at 25°C for 1 h and then washed with TBST and TBS, respectively. The membrane was stained with 10 mL of solution containing nitro blue tetrazolium (NBT) and 5-bromo-4-chloro-3-indolyl phosphate (BCIP) (Promega) in the dark. The reaction was terminated by adding double distilled water. The molecular masses of the polypeptides were estimated from a plot of the log of molecular mass of the marker standards versus the migration distance. Membranes were scanned and densitometer with a Bio-Rad Fluor-S Multimager and quantified using Bio-Rad Quantity One software in at least three independent blots.

### Statistical analysis

The experiment was arranged in a completely randomized design. Each sample time point for each treatment was comprised of three independent replicates. Data are plotted in figures as means ± standard deviations (S.D.). Least significant differences (LSD) were calculated to compare significant effects at the 5% level using DPS software (Zhejiang University, Version 3.0).

## Supporting Information

Figure S1 **Changes of fruit colour (A) and** ***FaNCED*** **expression (B) at different fruit ripening stages**. In (A), vertical bars represented standard deviations (SD) of means. Different letters indicated a statistical difference at the 5% level among data groups according to the Duncan's multiple range test. In (B), total RNA (10 μg per lane) was used for northern blot analysis and hybridized with DIG-labeled probe, and ethidium bromide-stained rRNA was shown as the loading control. The sequence of *FaNCED* was deposited in GenBank as JN006161.
(TIF)

Figure S2 **Changes of fruit colour after ABA treatment at the LG stage (about 15 days after post-anthesis (DPA)) (A) and at the W stage (about 23 DPA) (B).** Selected fruit were dipped for 1 min in a solution containing 0 (control) or 100 µM ABA, and then sampled at 0, 1, 4, 7 and 10 days. Vertical bars represented standard deviations (S.D.) of means. Different letters indicated a statistical difference at the 5% level among data groups according to the Duncan's multiple range test.
(TIF)

Figure S3 **Changes of** ***FaNCED*** **expression after ABA treatment at the LG stage (about 15 days after post-anthesis (DPA)) (A) and at the W stage (about 23 DPA) (B).** Selected fruit were dipped for 1 min in a solution containing 0 (control) or 100 µM ABA, and then sampled at 0, 2, 6, 24 and 48 hours. Total RNA (10 μg per lane) was used for northern blot analysis and hybridized with DIG-labeled probe, and ethidium bromide-stained rRNA was shown as the loading control.
(TIF)

## References

[1] Giovannoni JJ (2001). Molecular biology of fruit maturation and ripening.. Annu Rev Plant Physiol Plant Mol Biol.

[2] Giovannoni JJ (2004). Genetic regulation of fruit development and ripening.. Plant Cell.

[3] Alexander L, Grierson D (2002). Ethylene biosynthesis and action in tomato: a model for climacteric fruit ripening.. J Exp Bot.

[4] Manning K (1998). Isolation of a set of ripening-related genes from strawberry: their identification and possible relationship to fruit quality traits.. Planta.

[5] Chervin C, El-Kereamy A, Roustan JP, Latché A, Lamon J (2004). Ethylene seems required for the berry development and ripening in grape, a non-climacteric fruit.. Plant Sci.

[6] Lee S, Chung EJ, Joung YH, Choi D (2010). Non-climacteric fruit ripening in pepper: increased transcription of EIL-like genes normally regulated by ethylene.. Func Integr Genomics.

[7] Fei Z, Tang X, Alba RM, White JA, Ronning CM (2004). Comprehensive EST analysis of tomato and comparative genomics of fruit ripening.. Plant J.

[8] Lin ZF, Zhong SL, Grierson D (2009). Recent advances in ethylene research.. J Exp Bot.

[9] Li X, Xu CJ, Korba SS, Chen KS (2010). Regulatory mechanisms of textural changes in ripening fruits.. Cri Rev Plant Sci.

[10] Davies C, Boss PK, Robinson SP (1997). Treatment of grape berries, a nonclimacteric fruit with a synthetic auxin, retards ripening and alters the expression of developmentally regulated genes.. Plant Physiol.

[11] Nam YW, Tichit L, Leperlier M, Cuerq B, Marty I (1999). Isolation and characterization of mRNAs differentially expressed during ripening of wild strawberry (*Fragaria vesca L*.) fruits.. Plant Mol Biol.

[12] Aharoni A, Keizer LC, Van Den Broeck HC, Blanco-Portales R, Munoz-Blanco J (2002). Novel insight into vascular, stress, and auxin-dependent and -independent gene expression programs in strawberry, a nonclimacteric fruit.. Plant Physiol.

[13] Aharoni A, O'Connell AP (2002). Gene expression analysis of strawberry achene and receptacle maturation using DNA microarray.. J Exp Bot.

[14] Symons G, Davies C, Shavrukov Y, Dry I, Reid J (2006). Grapes on steroids. Brassinosteroids are involved in grape berry ripening.. Plant Physiol.

[15] Fu FQ, Mao WH, Shi K, Zhou YH, Asami T (2008). A role of brassinosteroids in early fruit development in cucumber.. J Exp Bot.

[16] Trainotti L, Pavanello A, Casadoro G (2005). Different ethylene receptors show an increased expression during the ripening of strawberries: does such an increment imply a role for ethylene in the ripening of these non-climacteric fruits?. J Exp Bot.

[17] Iannetta PPM, Laarhoven LJ, Medina-Escobar N, James EK, McManus MT (2006). Ethylene and carbon dioxide production by developing strawberries show a correlative pattern that is indicative of ripening climacteric fruit.. Physiol Planta.

[18] Mukkun L, Singh Z (2009). Methyl jasmonate plays a role in fruit ripening of ‘Pajaro’ strawberry through stimulation of ethylene biosynthesis.. Sci Hortic.

[19] Wheeler S, Loveys B, Ford C, Davies C (2009). The relationship between the expression of abscisic acid biosynthesis genes, accumulation of abscisic acid and the promotion of *Vitis vinifera* L. berry ripening by abscisic acid.. Aus J Grape Wine Res.

[20] Gambetta GA, Matthews MA, Shaghasi TH, McElrone AJ, Castellarin SD (2010). Sugar and abscisic acid signaling orthologs are activated at the onset of ripening in grape.. Planta.

[21] Kojima K (1996). Distribution and change of endogenous IAA and ABA in asparagus spear and orange fruit.. Chem Regul Plants.

[22] KondoS, Inoue K (1997). Abscisic acid and 1-aminocyclopropane-1-carboxylic acid (ACC) content during growth of ‘Satohnishiki’ cherry fruit, and the effect of ABA and ethephon application on fruit quality.. J Hortic Sci.

[23] Kondo S, Tomiyama A (1998). Changes of free and conjugated ABA in the fruit of Satohnishiki sweet cherry and the ABA metabolism after application of (s)-(+)-ABA.. J Hortic Sci Biotech.

[24] Zhang M, Yuan B, Leng P (2009). The role of ABA in triggering ethylene biosynthesis and ripening of tomato fruit.. J Exp Bot.

[25] Zhang M, Leng P, Zhang GL, Li XX (2009). Cloning and functional analysis of 9-cis-epoxycarotenoid dioxygenase (NCED) genes encoding a key enzyme during abscisic acid biosynthesis from peach and grape fruits.. J Plant Physiol.

[26] Finkelstein RR, Gampala SS, Rock CD (2002). Abscisic acid signaling in seeds and seedlings.. Plant Cell.

[27] Abe H, Urao T, Ito T, Seki M, Shinozaki K (2003). Arabidopsis AtMYC2 (bHLH) and AtMYB2 (MYB) function as transcriptional activators in abscisic acid signaling.. Plant Cell.

[28] González-García MP, Rodríguez D, Nicolás C, Rodríguez PL, Nicolás G (2003). Negative regulation of abscisic acid signaling by the *Fagus sylvatica* FsPP2C1 plays a role in seed dormancy regulation and promotion of seed germination.. Plant Physiol.

[29] Chernys JT, Zeevaart JAD (2000). Characterization of the 9-cis-epoxycarotenoid dioxygenase gene family and the regulation of abscisic acid biosynthesis in avocado.. Plant Physiol.

[30] Ban T, Ishimaru M, Kobayashi S, Goto-Yamamoto SN, Horiuchi S (2003). Abscisic acid and 2,4-dichlorophenoxyacetic acid affect the expression of anthocyanin biosynthetic pathway genes in ‘Kyoho’ grape berries.. J Hortic Sci Biotech.

[31] Jeong ST, Goto-Yamamoto N, Kobayashi S, Esaka M (2004). Effects of plant hormones and shading on the accumulation of anthocyanins and the expression of anthocyanin biosynthetic genes in grape berry skins.. Plant Sci.

[32] Lacampagne S, Gagné S, Gény L (2010). Involvement of abscisic acid in controlling the proanthocyanidin biosynthesis pathway in grape skin: new elements regarding the regulation of tannin composition and leucoanthocyanidin reductase (LAR) and anthocyanidin reductase (ANR) activities and expression.. J Plant Growth Regul.

[33] Giribaldi M, Gény L, Delrot S, Schubert A (2010). Proteomic analysis of the effects of ABA treatments on ripening *Vitis vinifera* berries.. J Exp Bot.

[34] Çakir B, Agasse A, Gaillard C, Saumonneau A, Deirot S (2003). A grape ASR protein involved in sugar and abscisic acid signaling.. Plant Cell.

[35] Schneider A, Salamini F, Gebhardt C (1997). Expression patterns and promoter activity of the cold-regulated gene *ci21A* of potato.. Plant Physiol.

[36] Huang JC, Lin SM, Wang CS (2000). A pollen-specific and desiccationassociated transcript in *Lilium longiflorum* during development and stress.. Plant Cell Physiol.

[37] Maskin L, Gubesblat GE, Moreno JE, Carrari FO, Frankel N (2001). Differential expression of the members of the *Asr* gene family in tomato (*Lycopersicon esculentum*).. Plant Sci.

[38] Hong SH, Kim IJ, Yang DC, Chung WI (2002). Characterization of an abscisic acid responsive gene homologue from *Cucumis melo*.. J Exp Bot.

[39] Jeanneau M, Gerentes D, Foueillassar X, Zivy M, Vidal J (2002). Improvement of drought tolerance in maize: towards the functional validation of the ZM-ASR1 gene and increase of water use efficiency by over-expressing C4-PEPC.. Biochimie.

[40] Kalifa Y, Perlson E, Gilad A, Konrad Z, Scolnik PA (2004). Over-expression of the water and salt stress-regulated *Asr1* gene confers an increased salt tolerance.. Plant Cell Environ.

[41] Yang CY, Chen YC, Jauh GY, Wang CS (2005). A lily ASR protein involves abscisic acid signaling and confers drought and salt resistance in Arabidopsis.. Plant Physiol.

[42] Liu HY, Dai JR, Feng DR, Liu B, Wang HB (2010). Characterization of a novel plantain *Asr* gene, *MpAsr*, that is regulated in response to infection of *Fusarium oxysporum* f. sp. cubense and abiotic stresses.. J Integr Plant Biol.

[43] Shkolnik D, Bar-Zvi D (2008). Tomato ASR1 abrogates the response to abscisic acid and glucose in Arabidopsis by competing with ABI4 for DNA binding.. Plant Biotechnol J.

[44] Iusem ND, Bartholomew DM, Hitz WD, Scolnik PA (1993). Tomato (*Lycopersicon esculentum*) transcript induced by water deficit and ripening.. Plant Physiol.

[45] Saumonneau A, Agasse A, Bidoyen MT, Lallemand M, Cantereau A (2008). Interaction of grape ASR proteins with a DREB transcription factor in the nucleus.. FEBS Letters.

[46] Zhang JJ, Wang X, Yu O, Tang JJ, Gu XG (2011). Metabolic profiling of strawberry (*Fragaria* × *ananassa* Duch.) during fruit development and maturation.. J Exp Bot.

[47] Perkins-Veazie P (1995). Growth and ripening of strawberry fruit.. Hortic Rev.

[48] Given NK, Venis MA, Grierson D (1988). Hormonal regulation of ripening in the strawberry, a nonclimacreric fruit.. Planta.

[49] Csukasi F, Osorio S, Gutierrez JR, Kitamura J, Giavalisco P (2011). Gibberellin biosynthesis and signalling during development of the strawberry receptacle.. http://dx.doi.org/10.1111/j.1469-8137.2011.03700.x.

[50] Manning K (1994). Changes in gene expression during strawberry fruit ripening and their regulation by auxin.. Planta.

[51] Civello PM, Powell ALT, Sabehat A, Bennet AB (1999). An expansin gene is expressed in ripening strawberry fruit.. Plant Physiol.

[52] Harpster MH, Brummell DA, Dunsmuir P (1998). Expression analysis of a ripening-specific, auxin-repressed endo-1,4-ß-glucanase gene in strawberry.. Plant Physiol.

[53] Tisza V, Kovács L, Balogh A, Heszky L, Kiss E (2010). Characterization of *FaSPT*, a SPATULA gene encoding a bHLH transcriptional factor from the non-climacteric strawberry fruit.. Plant Physiol Biochem.

[54] Shen GA, Pang YZ, Wu WS, Deng ZX, Liu XF (2005). Molecular cloning, characterization and expression of a novel *Asr* gene from *Ginkgo biloba*.. Plant Physiol Biochem.

[55] Canel C, Baiey-Serres JN, Roose ML (1995). Pummelo fruit transcript homologous to ripening-induced genes.. Plant Physiol.

[56] Padmanabhan V, Dias DMAL, Newton RJ (1997). Expression analysis of a gene family in loblolly pine (*Pinus taeda* L.) induced by water deficit stress.. Plant Mol Biol.

[57] Kalifa Y, Gilad A, Konrad Z, Zaccai M, Scolnik PA (2004). The water- and salt-stress regulated *Asr1* gene encodes a zinc-dependent DNA- binding protein.. Biochem J.

[58] Wang HJ, Hsu CM, Guang YJ, Wang CS (2005). A lily pollen ASR protein localizes to both cytoplasm and nuclei requiring a nuclear localization signal.. Physiol Planta.

[59] Liu DJ, Chen JY, Lu WJ (2011). Expression and regulation of the early auxin-responsive Aux/IAA genes during strawberry fruit development.. Mol Biol Rep.

[60] Frankel N, Carrari F, Hasson E, Iusem ND (2006). Evolutionary history of the Asr gene family.. Gene.

[61] Wang CS, Liau YE, Huang JC, Wu TD, Su CC (1998). Characterization of a desiccation-related protein in lily pollen during development and stress.. Plant Cell Physiol.

[62] Silhavy D, Hutvagner G, Barta E, Banfalvi Z (1995). Isolation and characterization of a water-stress-inducible cDNA clone from *Solanum chacoense*.. Plant Mol Biol.

[63] Riccardi F, Gazeau P, de Vienne D, Zivy M (1998). Protein changes in response to progressive water deficit in maize. Quantitative variation and polypeptide identification.. Plant Physiol.

[64] Vaidyanathan R, Kuruvilla S, Thomas G (1999). Characterization and expression pattern of an abscisic acid and osmotic stress responsive gene from rice.. Plant Sci.

[65] Yoneda Y (2000). Nucleoplasmic protein traffic and its significance to cell function.. Genes to Cells.

[66] Houde M, Daniel C, Lachapelle M, Allard F, Laliberte S (1995). Immunolocalization of freezing-tolerance-associated proteins in the cytoplasm and nucleoplasm of wheat crown tissues.. Plant J.

[67] Urtasun N, García SC, Iusem ND, Moretti MB (2010). Predominantly cytoplasmic localization in yeast of ASR1, a non-receptor transcription factor from plants.. The Open Biochem J.

[68] Konrad Z, Bar-Zvi D (2008). Synergism between the chaperone-like activity of the stress regulated ASR1 protein and the osmolyte glycine-betaine.. Planta.

[69] Frankel N, Nunes-Nesi A, Balbo I, Mazuch J, Centeno D (2007). ci21A/Asr1 expression influences glucose accumulation in potato tubers.. Plant Mol Biol.

[70] Jiang YM, Joyce DC (2003). ABA effects on ethylene production, PAL activity, anthocyanin and phenolic contents of strawberry fruit.. Plant Growth Regul.

[71] Kano Y, Asahira T (1981). Roles of cytokinin and abscisic acid in the maturing of strawberry fruit.. J Jap Soc Hortic Sci.

[72] Archbold DD (1988). Abscisic acid facilitates sucrose import by strawberry fruit explants and cortex disks in vitro.. HortSci.

[73] Pimentel P, Salvatierra A, Moya-León MA, Herrera P (2010). Isolation of genes differentially expressed during development and ripening of *Fragaria chiloensis* fruit by suppression subtractive hybridization.. J Plant Physiol.

[74] Griesser M, Vitzthum F, Fink B, Bellido ML, Raasch C (2008). Multi-substrate flavonol O-glucosyltransferases from strawberry (*Fragaria* × *ananassa*) achene and receptacle.. J Exp Bot.

[75] Fait A, Hanhineva K, Beleggia R, Dai N, Rogachev I (2008). Reconfiguration of the achene and receptacle metabolic networks during strawberry fruit development.. Plant Physiol.

[76] Archbold DD, Dennis FG (1984). Quantification of free ABA and free and conjugated. IAA in strawberry achene and receptacle tissue during fruit development. J Amer Soc Hort Sci.

[77] Martinez GA, Chaves AR, Anon MC (1994). Effect of gibberellic acid on ripening of strawberry fruit (*Fragaria* × *ananassa* Duch.).. J Plant Growth Regul.

[78] Zhu SH, Zhou J (2007). Effect of nitric oxide on ethylene production in strawberry fruit during storage.. Food Chem.

[79] Bustamante CA, Civello PM, Martínez GA (2009). Cloning of the promoter region of ß-xylosidase *(FaXyl1*) gene and effect of plant growth regulators on the expression of *FaXyl1* in strawberry fruit.. Plant Sci.

[80] Zhang FJ, Jin YJ, Xu XY, Lu RC, Chen HJ (2008). Study on the extraction, purification and quantification of jasmonic acid, abscisic acid and indole-3-acetic acid in plants.. Phytochem Anal.

[81] Wan CY, Wilkins TA (1994). A modified hot borate method significantly enhances the yield of high quality RNA from cotton (*Gossypium hirsutum L*.).. Anal Biochem.

[82] Mahdieh M, Mostajeran A (2009). Abscisic acid regulates root hydraulic conductance via aquaporin expression modulation in *Nicotiana tabacum*.. J Plant Physiol.

[83] Abel S, Theologis A (1994). Transient transformation of Arabidopsis leaf protoplasts: a versatile experimental system to study gene expression.. Plant J.

[84] Tian L, Kong WF, Pan QH, Zhan JC, Wen PF (2006). Expression of the chalcone synthase gene from grape and preparation of an anti-CHS antibody.. Protein Expres Purif.

[85] Saravanan SR, Rose JKC (2004). A critical evaluation of sample extraction techniques for enhanced proteomic analysis of recalcitrant plant tissues.. Proteomics.

