# Supplementary figures and images for: Molecular Characterization of a Strawberry FaASR Gene in Relation to Fruit Ripening
Source: PLoS One. 2011 Sep 6;6(9):e24649. doi: 10.1371/journal.pone.0024649 (PMC3167850; doi:10.1371/journal.pone.0024649)

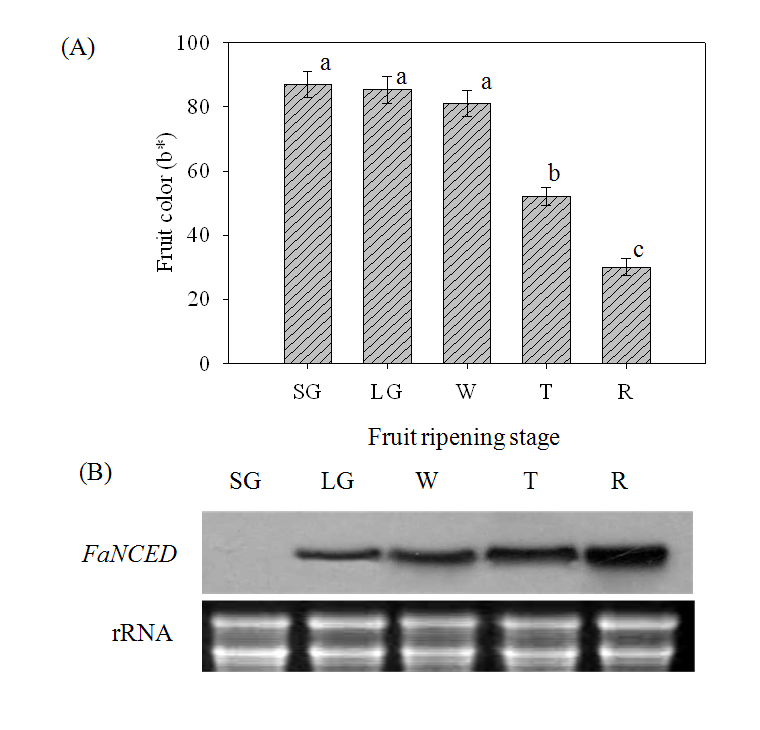

Supplement: Figure S1 — Changes of fruit colour (A) and FaNCED expression (B) at different fruit ripening stages. In (A), vertical bars represented standard deviations (SD) of means. Different letters indicated a statistical difference at the 5% level among data groups according to the Duncan's multiple range test. In (B), total RNA (10 μg per lane) was used for northern blot analysis and hybridized with DIG-labeled probe, and ethidium bromide-stained rRNA was shown as the loading control. The sequence of FaNCED was deposited in GenBank as JN006161. (TIF) [file pone.0024649.s001.tif]

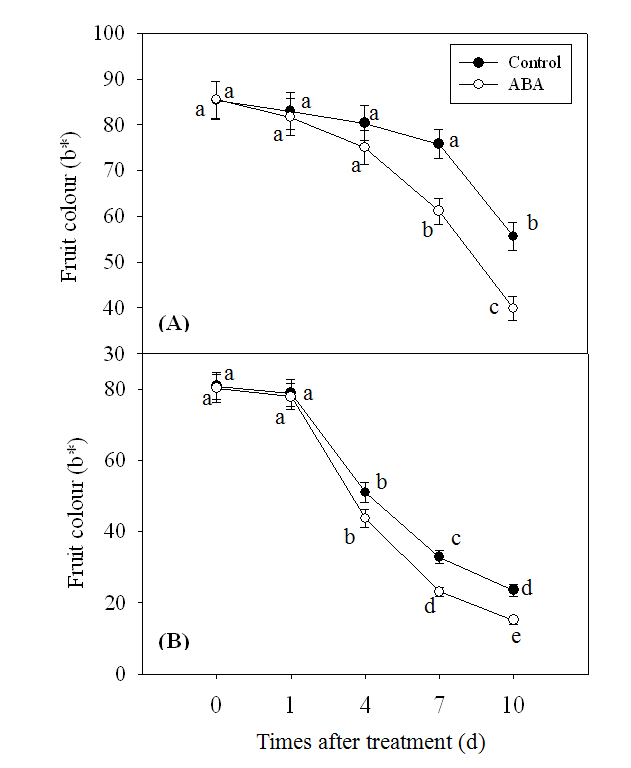

Supplement: Figure S2 — Changes of fruit colour after ABA treatment at the LG stage (about 15 days after post-anthesis (DPA)) (A) and at the W stage (about 23 DPA) (B). Selected fruit were dipped for 1 min in a solution containing 0 (control) or 100 µM ABA, and then sampled at 0, 1, 4, 7 and 10 days. Vertical bars represented standard deviations (S.D.) of means. Different letters indicated a statistical difference at the 5% level among data groups according to the Duncan's multiple range test. (TIF) [file pone.0024649.s002.tif]

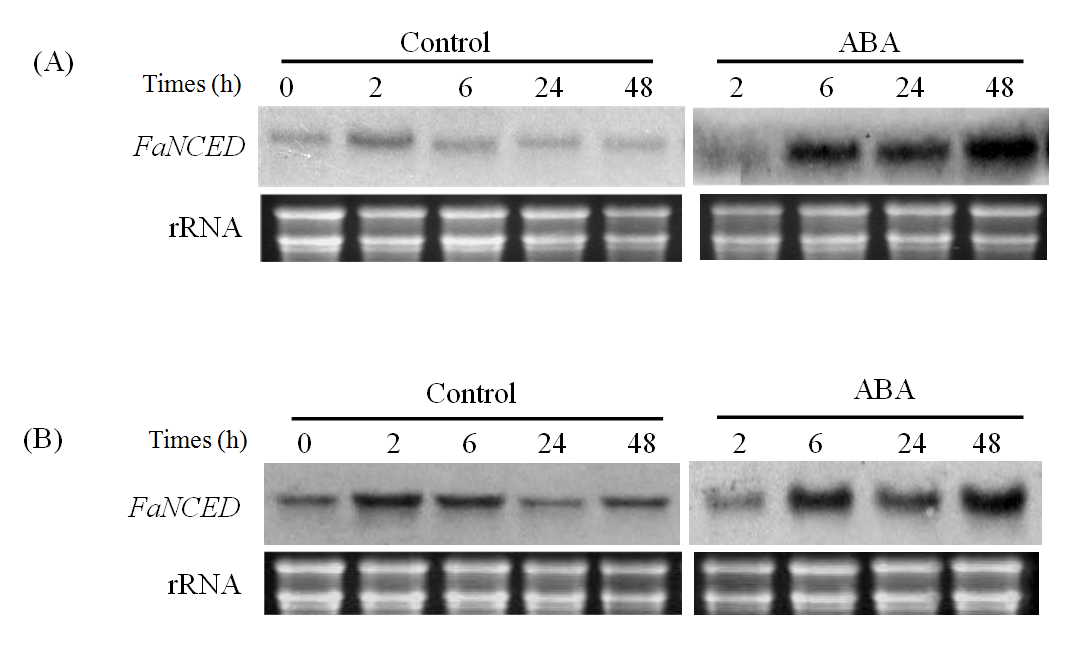

Supplement: Figure S3 — Changes of FaNCED expression after ABA treatment at the LG stage (about 15 days after post-anthesis (DPA)) (A) and at the W stage (about 23 DPA) (B). Selected fruit were dipped for 1 min in a solution containing 0 (control) or 100 µM ABA, and then sampled at 0, 2, 6, 24 and 48 hours. Total RNA (10 μg per lane) was used for northern blot analysis and hybridized with DIG-labeled probe, and ethidium bromide-stained rRNA was shown as the loading control. (TIF) [file pone.0024649.s003.tif]
